# Plasma 8-Hydroxy-2′-Deoxyguanosine Levels in Patients with Type 2 Diabetes Mellitus

**DOI:** 10.3390/diagnostics16213435

**Published:** 2026-10-22

**Authors:** Karina-Alexandra Cojocaru, Mariana Pavel-Tanasa, Oana Roxana Oprea, Larisa Ghemiș, Delia Reurean-Pintilei, Andra Melissa Tarțău, Iris Bararu-Bojan, Georgeta Liliana Foia

**Affiliations:** 1 Grigore T. Popa University of Medicine and Pharmacy Iasi, 700115 Iasi, Romania; karina-alexandra.cojocaru@d.umfiasi.ro (K.-A.C.); mariana.pavel-tanasa@umfiasi.ro (M.P.-T.); entuc.andra-melissa@d.umfiasi.ro (A.M.T.); iris.bararu@umfiasi.ro (I.B.-B.); georgeta.foia@umfiasi.ro (G.L.F.); 2 George Emil Palade University of Medicine Pharmacy Science and Technology of Targu Mures, 540139 Târgu Mureș, Romania; oana.oprea@umfst.ro; 3 University “Ștefan cel Mare”, 720229 Suceava, Romania; drdeliapintilei@gmail.com

**Keywords:** 8-OHdG, diabetes, ROS

## Abstract

**Background/Objectives**: Type 2 diabetes mellitus (T2DM) is a complex metabolic disorder characterized not only by impaired carbohydrate metabolism but also by alterations in lipid and protein metabolism. Hyperglycaemia in T2DM can promote the excessive production of reactive oxygen species (ROS), leading to oxidative stress. 8-Hydroxy-2′-deoxyguanosine (8-OHdG) is a biomarker of oxidative DNA damage commonly used to investigate oxidative stress. **Methods**: In this cross-sectional analytical observational study, patients diagnosed with T2DM and healthy controls were enrolled. Plasma 8-OHdG concentrations were measured using an enzyme-linked immunosorbent assay (ELISA). Associations between 8-OHdG concentrations and age, fasting blood glucose, and lipid profile parameters were evaluated. These associations were further explored in patient subgroups defined according to glycated haemoglobin (HbA1c) levels. **Results**: Patients with T2DM had higher plasma 8-OHdG concentrations than healthy controls (Cohen’s d = 1.092; 95% CI, 0.646–1.532; *p* < 0.001). After adjustment for age, the between-group difference was attenuated, with an adjusted mean difference of 28.84 ng/mL (95% CI, 0.278–57.41; *p* = 0.048). In the overall cohort, statistically significant correlations were identified between 8-OHdG levels and age (ρ = 0.379; 95% CI, 0.164–0.556; *p* < 0.001), fasting blood glucose (ρ = 0.266; 95% CI, 0.059–0.474; *p* = 0.011), and triglycerides (ρ = 0.376, 95% CI, 0.178–0.554; *p* < 0.001). Stratification of patients according to HbA1c levels revealed no statistically significant differences in 8-OHdG concentrations among the subgroups. Furthermore, none of the correlations examined within these subgroups remained statistically significant after adjustment for multiple testing. **Conclusions**: Plasma 8-OHdG concentrations were higher in patients with T2DM than in healthy controls, although the magnitude of the between-group difference was attenuated after adjustment for age. In the overall study population, 8-OHdG concentrations were associated with age, fasting blood glucose, and triglyceride concentrations. These findings suggest that 8-OHdG levels may be associated with demographic and metabolic factors. Given the cross-sectional design, these associations should not be interpreted as causal. Further prospective studies with larger cohorts are needed to evaluate the potential clinical relevance of plasma 8-OHdG measurement in T2DM.

## 1. Introduction

Type 2 diabetes mellitus (T2DM) is a complex, chronic metabolic disorder, characterized by impaired insulin action and/or reduced insulin secretion, which leads to increased plasma glucose concentrations [1]. Chronic hyperglycaemia in T2DM can promote excessive production of reactive oxygen species (ROS) through several mechanisms, including glucose auto-oxidation, increased formation of advanced glycation end products, activation of the polyol pathway, and mitochondrial dysfunction [1]. These reactive species are also produced endogenously by normal cellular metabolism and play an important role in maintaining cellular homeostasis. Additionally, exogenous sources such as ionizing radiation, chemicals, alcohol, tobacco, and infectious agents can also contribute to ROS production [2,3]. Living organisms are endowed with natural antioxidant systems capable of neutralizing excessive ROS [3]. However, an imbalance between ROS production and antioxidant defences induces oxidative stress [4]. Therefore, ROS can damage lipids, nucleic acids, and proteins, leading to cellular dysfunction [2].

Biomarkers of oxidative stress can be important tools in assessing disease progression. Several methods have been developed to assess oxidative stress, including direct measurement of ROS, oxidative damage, and antioxidant status [3].

Among oxidative stress biomarkers, 8-hydroxy-2′-deoxyguanosine (8-OHdG) is one of the most extensively studied because it reflects oxidative modifications to DNA [5]. 8-OHdG and its tautomer, 8-oxo-7,8-dihydro-2′-deoxyguanosine (8-oxo-dG), are generated by the oxidation of guanine and represent stable end products of oxidative DNA damage. In the literature, the terms 8-OHdG and 8-oxo-dG are frequently used to describe the dynamic process of free radical-induced oxidative stress [6]. Although more than 20 oxidative DNA lesions have been identified, guanine is particularly susceptible to oxidative damage because it has a lower oxidation potential than other nucleobases, such as adenine (A), thymine (T), and cytosine (C) [6,7].

To date, 8-OHdG has been among the most extensively researched products of oxidative DNA damage, and it has been investigated as a biomarker of both oxidative DNA damage and carcinogenesis [8,9]. Due to its biological significance, numerous studies have focused on measuring 8-OHdG in blood, urine, DNA, or various types of tissues. The quantification of this biomarker, although intensely debated, is performed by highly sensitive methods, such as liquid or gas chromatography coupled with mass spectrometry and electrochemical detection, as well as enzyme-linked immunosorbent assays (ELISAs) [7,10].

Assessing 8-OHdG levels in patients with T2DM may provide insights into oxidative DNA damage. 8-OHdG may contribute to a better understanding of individual metabolic alterations, but further studies are required to establish its potential clinical utility. Therefore, the present study aimed to compare 8-OHdG concentrations in patients with T2DM and healthy controls. Additionally, associations between 8-OHdG concentrations and age, fasting blood glucose levels, and lipid profile parameters were explored both in the overall study population and in subgroups defined by HbA1c levels.

## 2. Materials and Methods

### 2.1. Study Design, Setting, and Participants

In this cross-sectional, analytical observational study, 100 patients were enrolled, 50 diagnosed with T2DM (study group) and 50 healthy subjects (control group). Participants were recruited from the Municipal Emergency Hospital Roman between September 2024 and December 2024. All procedures were conducted in accordance with the Declaration of Helsinki. The study protocol was approved by the Ethics Committee of Grigore T. Popa University of Medicine and Pharmacy Iași (approval No. 341/11 August 2023) and the Ethics Committee of the Municipal Emergency Hospital Roman (approval No. 32/17 September 2024). All participants were included in the study after providing written informed consent.

The inclusion criteria for the T2DM group were as follows: adults (>18 years) with a physician-confirmed diagnosis of T2DM for at least 6 months. Subjects who refused inclusion in the study, those with type 1 diabetes, or those with associated comorbidities that made clinical examination impossible were excluded.

The control group consisted of healthy individuals without a history of diabetes mellitus or other metabolic diseases. Of the 50 subjects initially assessed for eligibility, 9 were excluded because their triglyceride levels exceeded 200 mg/dL and/or their total cholesterol levels exceeded 240 mg/dL. These thresholds were used to exclude participants with relevant lipid abnormalities and to ensure that the control group consisted of individuals without significant metabolic alterations.

### 2.2. Clinical and Laboratory Parameters

For all selected subjects, venous blood was collected after an overnight fast. Samples were collected in tubes without anticoagulant and tubes containing ethylenediaminetetraacetic acid (EDTA) (BD Vacutainer, Becton, Dickinson, Franklin Lakes, NJ, USA). Serum and plasma were separated by centrifugation at 2000× *g* for 10 min at room temperature. Serum samples were allowed to clot at room temperature for 30 min before centrifugation. Aliquots were stored at −80 °C until analysis. Prior to plasma 8-OHdG assessment, aliquots were thawed and homogenized using a vortex mixer.

Fasting blood glucose (G), triglycerides (TG), total cholesterol (TC), HDL-cholesterol (HDL-C), and LDL-cholesterol (LDL-C) were measured using the Beckman Coulter AU480 (Beckman Coulter, CA, USA) and the Cobas Pro (Roche Diagnostics, Rotkreuz, Switzerland). The estimated glomerular filtration rate (eGFR) was calculated using the 2009 CKD-EPI creatinine equation, based on standardized serum creatinine, age, and sex, expressed in mL/min/1.73 m^2^. HbA1c was measured by high-performance liquid chromatography (HPLC) using the Arkray Adams A1c Lite analyser (ARKRAY, Kyoto, Japan).

Plasma 8-OHdG concentrations were measured using a competitive ELISA technique (E-EL-0028, Elabscience, Houston, TX, USA), according to the manufacturer’s protocol. The assay had a detection range of 1.56–100 ng/mL and a sensitivity of 0.94 ng/mL. The manufacturer reports intra-assay and inter-assay coefficients of variation of <10% and a recovery range of 80–120%. A sample volume of 50 μL was used, and optical density (OD) was measured spectrophotometrically at 450 ± 2 nm. The concentration of 8-OHdG in the tested samples was calculated based on the corresponding OD values and the standard curve. 8-OHdG measurements were performed in duplicate, and the mean value was subsequently used in the statistical analysis. Samples yielding values above the upper limit of the standard curve (100 ng/mL) (*n* = 21) were diluted and reanalysed to ensure that the measurements fell within the assay’s measurement range (1.56–100 ng/mL). Final concentrations were calculated by multiplying the concentration obtained from the standard curve by the corresponding dilution factor.

### 2.3. Statistical Analysis

Statistical processing of the data was performed using Microsoft Excel and SPSS version 31.0. Variables were summarized as mean ± standard deviation (SD) or median (interquartile range (IQR)). Data distribution was evaluated using the Kolmogorov–Smirnov and Shapiro–Wilk tests.

Comparative analysis of quantitative variables was performed using Student’s *t*-test, Welch’s *t*-test, Mann–Whitney U test, or Kruskal–Wallis test, based on data distribution and homogeneity of variances. The choice between Student’s *t*-test and Welch’s *t*-test was based on Levene’s test for equality of variances: Student’s *t*-test was used when variances were homogeneous (Levene’s *p* ≥ 0.05), whereas Welch’s *t*-test was used when this assumption of equal variances was not met (Levene’s *p* < 0.05). When the assumption of normality was not met, the Mann–Whitney U test was performed as a non-parametric analysis. For comparisons performed using Student’s *t*-test, Cohen’s *d* was calculated as a measure of effect size: negligible effect (d < 0.2), small effect (0.2 ≤ d < 0.5), medium (0.5 ≤ d < 0.8), and large (d ≥ 0.8). For the Kruskal–Wallis test, the effect size was interpreted according to the following thresholds: negligible effect (η^2^ < 0.01), small (η^2^ < 0.06), medium (η^2^ < 0.14), and large (η^2^ > 0.14). Additionally, Spearman’s rank correlation coefficient (ρ) was calculated to assess associations between continuous variables. Correlations were interpreted as negligible (ρ ≤ 0.25), weak (0.25 < ρ ≤ 0.5), moderate (0.5 < ρ ≤ 0.75), and strong (ρ > 0.75). *p*-values for Spearman’s correlation coefficients were calculated using an asymptotic test. The 95% confidence intervals (CIs) for correlation coefficients were estimated using bootstrap resampling with the bias-corrected and accelerated (BCa) method, based on 1000 bootstrap resamples.

For the exploratory analyses stratified by HbA1c level, the Benjamini–Hochberg procedure was applied to control the false discovery rate (FDR) across the entire family of Spearman correlation tests, comprising the associations between plasma 8-OHdG concentrations and clinical and biochemical variables within each of the three HbA1c subgroups. Both unadjusted *p*-values and Benjamini–Hochberg-adjusted *p*-values (q-values) were considered in the interpretation of these exploratory analyses.

An age-adjusted analysis of covariance (ANCOVA) was performed to assess the association between T2DM status and plasma 8-OHdG concentrations, with plasma 8-OHdG concentration as the dependent variable, T2DM status as the fixed factor, and age as the covariate. The homogeneity of regression slopes assumption was assessed by testing the T2DM status × age interaction.

Receiver operating characteristic (ROC) curve analysis was performed to explore the discriminatory ability of plasma 8-OHdG for distinguishing participants with T2DM from controls. The area under the curve (AUC), sensitivity, specificity, positive and negative predictive values, and overall accuracy were calculated. The optimal cut-off value was determined using Youden’s index.

Statistical significance was considered at *p* < 0.05.

## 3. Results

### 3.1. 8-OHdG Values in the T2DM and Control Groups

The study included 50 patients diagnosed with T2DM and 41 healthy subjects (controls). Out of 91 subjects included in the study, 34 (37.4%) were male, and the mean age was 60 years (range, 45–83 years). The mean (±SD) plasma 8-OHdG concentration was 70.5 ± 50.4 ng/mL (range, 12.2–216.7 ng/mL). There were no significant differences between men and women (69.7 ± 56.01 ng/mL versus 71.02 ± 47.26 ng/mL). The biochemical characteristics of the study population are summarized in Table 1.

Plasma 8-OHdG concentrations were compared between patients with T2DM and healthy controls. The mean 8-OHdG concentration in the T2DM group was 92.41 ± 58.04 ng/mL (median, 88.63 ng/mL), whereas the control group had a mean concentration of 43.90 ± 16.26 ng/mL (median, 41.52 ng/mL). The difference between the two groups was statistically significant (*p* < 0.001), with a large unadjusted standardized effect size (Cohen’s d = 1.092; 95% CI, 0.646–1.532) (Table 2). The Mann–Whitney U test was additionally performed as a non-parametric sensitivity analysis. The results were concordant with those of Welch’s *t*-test, confirming the consistency of the observed between-group difference (U = 571.000, *p* < 0.001, Z = −3.621, r = 0.38).

Correlations between 8-OHdG concentrations and clinical and biochemical parameters were subsequently assessed. In the overall study population, 8-OHdG concentrations showed weak but statistically significant positive correlations with age (ρ = 0.379, *p* < 0.001), fasting blood glucose (ρ = 0.266, *p* = 0.011), and triglyceride levels (ρ = 0.376, *p* < 0.001). The estimated correlation coefficients had limited precision due to the wide confidence intervals. No statistically significant correlations were observed between 8-OHdG concentrations and total cholesterol (ρ = −0.175, *p* = 0.097), LDL-cholesterol (ρ = −0.181, *p* = 0.086), or HDL-cholesterol (ρ = −0.171, *p* = 0.106), although the coefficients indicated weak negative correlations.

Within the T2DM group, 8-OHdG concentrations showed a weak positive correlation with triglyceride levels (ρ = 0.300, *p* = 0.034, 95% bootstrap CI: −0.027 to 0.613). The confidence interval included zero, indicating uncertainty in the estimated correlation; therefore, this association should be interpreted cautiously. Positive correlations with age (ρ = 0.233, *p* = 0.104) and negative correlation with HDL-cholesterol (ρ = −0.119, *p* = 0.409) were also observed; however, these associations did not reach statistical significance (Table 3).

Given the significant age difference between groups and the observed association between age and plasma 8-OHdG concentrations, an age-adjusted ANCOVA was performed, with plasma 8-OHdG concentration as the dependent variable, T2DM status as the fixed factor, and age as the covariate. The homogeneity of regression slopes was assessed by testing the T2DM status × age interaction (F = 1.314, *p* = 0.255, partial η^2^ = 0.015). The Shapiro–Wilk test did not indicate a significant departure from normality of the residuals (W = 0.981, *p* = 0.191), and visual inspection of the Q–Q plot was consistent with this finding. In the model without the interaction term, the group effect remained statistically significant after adjustment for age, although the effect was attenuated (F = 4.03, *p* = 0.048, partial η^2^ = 0.044). The estimated marginal mean 8-OHdG concentration was 54.7 ng/mL (SE = 9.14; 95% CI, 36.5–72.9) in the control group and 83.5 ng/mL (SE = 7.95; 95% CI, 67.7–99.3) in the T2DM group. The adjusted between-group difference was 28.84 ng/mL (95% CI, 0.278–57.41; *p* = 0.048). Compared with the unadjusted difference of 48.51 ng/mL, the adjusted difference of 28.84 ng/mL was approximately 40% smaller.

**Figure 1:**
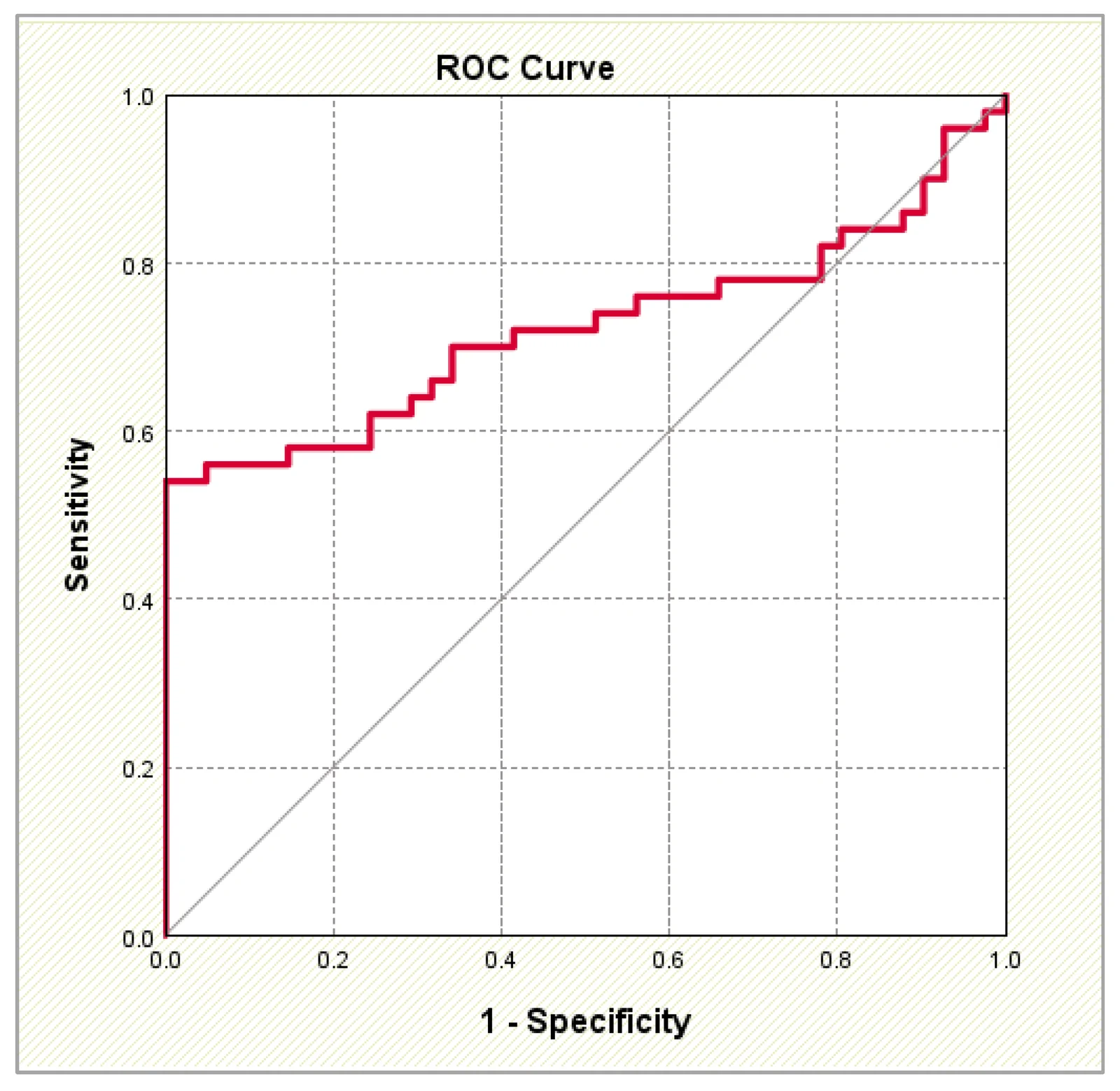
Receiver operating characteristic (ROC) curve assessing the ability of plasma 8-OHdG to discriminate between patients with T2DM and healthy controls. The area under the ROC curve (AUC) was 0.722 (95% CI, 0.614–0.831; *p* < 0.001).

To further explore the discriminatory performance of plasma 8-OHdG, receiver operating characteristic (ROC) curve analysis was performed to assess its ability to distinguish patients with T2DM from healthy controls. The area under the ROC curve (AUC) was 0.722 (95% CI, 0.614–0.831; *p* < 0.001), indicating moderate discriminatory ability. The optimal cut-off value, determined using Youden’s index, was 80.5 ng/mL, yielding a sensitivity of 54.00% (95% CI, 39.32–68.19%) and a specificity of 100.00% (95% CI, 91.40–100.00%). At this threshold, the positive predictive value was 100.00%, the negative predictive value was 64.06% (95% CI, 56.90–70.65%), and the overall accuracy was 74.7% (95% CI, 64.53–83.25%) (Figure 1).

### 3.2. 8-OHdG Analysis According to HbA1c Categories

In an exploratory post hoc analysis, patients with T2DM were stratified into three subgroups according to HbA1c levels (<7%, 7–8%, and >8%). No statistically significant differences in 8-OHdG concentrations were observed among the subgroups.

The lowest 8-OHdG concentrations were observed in patients with HbA1c levels of 7–8%, with a mean concentration of 79.81 ± 54.15 ng/mL (median, 66.96 ng/mL; IQR, 33.14–130.43). Patients with HbA1c levels < 7% had a mean concentration of 89.49 ± 58.38 ng/mL (median, 80.99 ng/mL; IQR, 31.86–142.8), whereas the highest concentrations were observed in patients with HbA1c levels > 8% (mean, 105.86 ± 61.24 ng/mL; median, 95.76 ng/mL; IQR, 49.87–162.42). However, the overall comparison among the HbA1c subgroups was not statistically significant (η^2^ < 0.01, *p* = 0.535), and pairwise comparisons likewise revealed no statistically significant differences.

Clinical and biochemical parameters were subsequently evaluated within each HbA1c subgroup. Before adjustment for multiple comparisons, several nominally significant correlations were observed. In patients with HbA1c levels < 7%, 8-OHdG concentrations were positively correlated with triglyceride levels (ρ = 0.551, *p* = 0.008) and total cholesterol levels (ρ = 0.446, *p* = 0.038). In patients with HbA1c levels of 7–8%, a positive correlation was observed between 8-OHdG concentrations and age (ρ = 0.625, *p* = 0.030), while a negative correlation was observed with HDL-cholesterol levels (ρ = −0.575, *p* = 0.050). In the HbA1c > 8% subgroup, 8-OHdG concentrations were negatively correlated with total cholesterol levels (ρ = −0.538, *p* = 0.031) (Table 4).

However, none of these correlations remained statistically significant after adjustment for multiple comparisons using the Benjamini–Hochberg procedure. Therefore, these subgroup findings should be considered exploratory and interpreted with caution.

Overall, plasma 8-OHdG concentrations were higher in patients with T2DM than in controls, although the between-group difference was attenuated after adjustment for age. 8-OHdG concentrations were positively correlated with age, fasting blood glucose, and triglyceride levels. However, stratification according to HbA1c categories did not reveal significant differences in 8-OHdG concentrations between the subgroups.

## 4. Discussion

Diabetes mellitus is a metabolic disorder associated with increased oxidative stress. The maintenance of genomic stability is essential for normal cellular functions; however, excessive oxidative stress can damage DNA, including through the oxidative modification of nitrogenous bases [11,12]. Among the biomarkers of oxidative DNA damage, 8-OHdG is one of the most extensively studied. It is formed through hydroxylation of 2′-deoxyguanosine. During DNA repair, oxidized guanine bases can be removed from DNA and subsequently detected in biological fluids, including blood and urine [13]. Therefore, measurement of 8-OHdG concentrations in patient serum, plasma, or urine may provide an indication of oxidative DNA damage [3].

The present study was designed to investigate plasma 8-OHdG concentrations in patients with T2DM and to explore their relationship with glycaemic parameters. Plasma 8-OHdG concentrations were higher in patients with T2DM than in healthy subjects, consistent with previous studies reporting increased concentrations of oxidative biomarkers in patients with diabetes. Mukhtar et al. reported significantly higher serum 8-OHdG levels in patients with diabetes than in controls [14]. Similarly, Gulbay and Kaya found increased 8-OHdG concentrations in patients with T2DM and dyslipidaemia (characterized by elevated total cholesterol, LDL-cholesterol, triglycerides, and/or reduced HDL-cholesterol levels) compared with patients with T2DM without dyslipidaemia and healthy subjects [11]. Additionally, significantly elevated serum 8-OHdG concentrations have been observed in newly diagnosed patients with T2DM compared with healthy subjects, supporting an association between oxidative stress and diabetes [15].

However, in our study, the T2DM group was substantially older than the control group, and age was positively associated with plasma 8-OHdG concentrations in the overall study population. After adjustment for age, the between-group difference decreased by approximately 40%. This attenuation suggests that age may have contributed to the observed difference. Given the age imbalance between groups and the possibility of residual confounding, the magnitude of the between-group difference should be interpreted cautiously.

To further assess the ability of plasma 8-OHdG concentrations to distinguish patients with T2DM from healthy controls, ROC curve analysis was performed. The resulting area under the curve (AUC) was 0.722, indicating moderate discriminatory ability. This indicates that, for a randomly selected pair consisting of one patient with T2DM and one control, there is approximately a 72.2% probability that the patient with diabetes will have a higher 8-OHdG concentration than the control. Thus, although the ROC analysis showed statistically significant discriminatory performance, the moderate AUC indicates that its ability to distinguish between the two groups is limited. Therefore, although plasma 8-OHdG may reflect increased oxidative stress associated with T2DM, its moderate discriminatory performance does not support its use as a standalone diagnostic biomarker. The ROC findings should be considered exploratory and require independent validation before any clinical diagnostic implications can be drawn.

The biological significance of these findings should also be considered in the context of oxidative stress associated with T2DM. Diabetes mellitus affects not only carbohydrate metabolism but also protein and lipid metabolism. Chronic hyperglycaemia is associated with increased oxidative stress, resulting from an imbalance between the production of reactive oxygen species and the antioxidant defence system. Due to the instability of ROS, oxidative stress is commonly assessed by measuring different biomarkers, including 8-OHdG [16]. Among the nitrogenous bases in nucleic acids, guanine is particularly susceptible to oxidative damage induced by ROS [17,18]. In their study, Al-Aubaidy and Jelinek observed significantly higher 8-OHdG levels in both diabetic and prediabetic patients compared with the healthy control group, suggesting that this biomarker may be useful for the early detection of oxidative damage associated with metabolic dysfunction. Furthermore, elevated 8-OHdG levels have been associated with oxidative DNA damage in tissue and vascular compartments, which may be related to an increased risk of cardiovascular complications [19]. Beyond vascular complications, oxidative DNA damage has also been involved in the neurological manifestations of T2DM. Huang et al. also reported an association between serum 8-OHdG concentrations, adiponectin levels, and cognitive status. Elevated 8-OHdG levels, together with decreased adiponectin concentrations, may contribute to cognitive decline in elderly patients with T2DM [20]. These results are further supported by a recent experimental study in mice fed a high-fat diet, which reported increased 8-OHdG levels in the hippocampus, accompanied by impaired cognitive function, further supporting a potential link between oxidative stress and metabolic dysfunction associated with cognitive impairment [21].

These findings shed light on the potential clinical relevance of 8-OHdG as a biomarker of oxidative DNA damage. Understanding the mechanisms responsible for its formation and accumulation is essential for interpreting its clinical significance. In addition to increased oxidative stress, the accumulation of oxidative DNA damage may also result from alterations in DNA repair mechanisms. One of the key enzymes involved in the repair of oxidized DNA bases is MUTYH, a component of the base excision repair pathway. The AluYb8 polymorphism of the MUTYH gene has been associated with increased 8-OHdG concentrations and has been proposed as a potential genetic factor related to T2DM susceptibility [22]. These data indicate that both enhanced oxidative stress and impaired DNA repair may contribute to oxidative DNA damage in T2DM. It should also be considered that 8-OHdG concentrations may be influenced by several physiological and environmental factors. Ageing, tobacco smoking, and occupational exposure to chemical, physical, or biological agents have been associated with higher 8-OHdG levels [8,23,24].

However, data concerning the relationship between 8-OHdG and metabolic parameters remain inconsistent, highlighting the need for further targeted investigations to better characterize the interplay between oxidative DNA damage and systemic metabolic factors. In the present study, plasma 8-OHdG concentrations showed positive correlations with age, fasting blood glucose, and triglyceride levels in the overall study population. Among patients with T2DM, a weak positive correlation was also observed between plasma 8-OHdG concentrations and triglyceride levels; however, the uncertainty reflected by the bootstrap confidence interval warrants cautious interpretation. These findings are compatible with a potential association between oxidative DNA damage and lipid metabolism, although the subgroup analysis does not provide conclusive evidence of this relationship.

Previous studies have also reported associations between 8-OHdG concentrations and lipid parameters. Gulbay and Kaya highlighted positive correlations between 8-OHdG and triglycerides, total cholesterol, and LDL cholesterol [11]. In contrast, Kikuchi et al. reported negative associations between urinary 8-OHdG concentrations and total cholesterol levels after multivariate adjustment. In subgroup analyses, a significant inverse association with LDL-cholesterol was observed only in women younger than 48 years. These associations varied according to patient characteristics, including sex and smoking status, highlighting the complex relationship between oxidative stress, lipid metabolism, and individual patient characteristics [25]. The variability in these findings may reflect differences in the biological sample analysed, study populations, and analytical approaches, as well as the multifactorial nature of oxidative stress in T2DM.

In addition to lipid metabolism, age appears to be another important factor associated with oxidative DNA damage. In the present study, plasma 8-OHdG concentrations were positively correlated with age in the overall study population, suggesting that oxidative DNA damage may be associated with advancing age. However, this association was not statistically significant when the analysis was restricted to patients with T2DM. Notably, the mean age of the healthy control group was approximately 16 years lower than that of patients with T2DM, raising the possibility that age may have acted as a confounding factor in the comparison between groups. To assess the potential influence of this age difference on the comparison of plasma 8-OHdG concentrations between patients with T2DM and healthy controls, an age-adjusted analysis of covariance (ANCOVA) was performed. After adjustment for age, the difference in plasma 8-OHdG concentrations between patients with T2DM and healthy controls was attenuated by approximately 40%, although it remained nominally statistically significant. This reduction suggests that age may have contributed to the observed difference in plasma 8-OHdG concentrations. Nevertheless, given the marked age imbalance between groups and the possibility of residual confounding, these findings should be interpreted cautiously.

The potential contribution of age to oxidative DNA damage is also supported by previous research. Rogulj et al. reported higher urinary 8-OHdG concentrations in older patients with T2DM and metabolic syndrome, suggesting a possible contribution of age to oxidative DNA damage in the context of metabolic disease [26]. In the study by Gan et al., urinary levels of 8-OHdG and 8-oxo-7,8-dihydroguanosine (8-oxoGsn) correlated with age in healthy individuals aged 21–90 years, with a stronger correlation observed for 8-oxoGsn [27]. Liu et al. reported a positive association between age and urinary 8-OHdG levels in healthy individuals, whereas the correlation did not reach statistical significance in patients with T2DM [28]. Similarly, Dong et al. found no correlation between age and urinary 8-OHdG levels in 60 patients with T2DM, although these levels were higher in patients than in controls and correlated significantly with HbA1c [29]. However, these studies assessed urinary 8-OHdG, whereas our study measured plasma 8-OHdG, limiting direct comparisons with our findings. More directly relevant to our study, Mahmoud et al. measured serum 8-OHdG in both healthy individuals and patients with T2DM and found no statistically significant correlation with age in either group [30].

Taken together, these findings suggest that the association between age and 8-OHdG concentrations may vary according to the study population and biological sample analysed. In our study, age was positively correlated with plasma 8-OHdG concentrations in the overall population. Furthermore, adjustment for age attenuated the difference in plasma 8-OHdG concentrations between patients with T2DM and healthy controls, although this difference remained statistically significant.

Beyond the influence of age, hyperglycaemia is considered one of the major contributors to oxidative stress in T2DM. In the present study, plasma 8-OHdG concentrations were positively correlated with fasting blood glucose levels. Consistent with our findings, Al-Aubaidy and Jelinek reported progressively higher serum 8-OHdG concentrations from healthy individuals to subjects with prediabetes and patients with T2DM, supporting an association between altered glucose metabolism and oxidative DNA damage. The authors observed elevated 8-OHdG concentrations even in individuals with prediabetes despite relatively normal HbA1c values [19]. These observations suggest that the mechanisms linking glycaemic dysregulation and oxidative DNA damage involve several interconnected pathways that may not be fully captured by HbA1c levels alone. In this context, Spoto et al. reported higher serum 8-OHdG concentrations in patients with diabetes-related kidney disease than in diabetic subjects without renal complications. Furthermore, their analysis showed that the association between 8-OHdG concentrations and renal outcomes varied according to HbA1c levels [31].

Although fasting blood glucose was positively associated with plasma 8-OHdG concentrations in our study, this association was not reflected in the analyses based on HbA1c-defined subgroups. No statistically significant differences in plasma 8-OHdG concentrations were observed across the HbA1c categories, although the relatively small number of patients in each category may have limited the ability to detect modest differences. Before adjustment for multiple comparisons, several nominal correlations were observed within the HbA1c subgroups. In patients with HbA1c levels below 7%, plasma 8-OHdG concentrations were positively correlated with triglyceride and total cholesterol levels. In the subgroup with HbA1c values between 7% and 8%, plasma 8-OHdG concentrations were positively correlated with age and negatively correlated with HDL-cholesterol levels. In patients with HbA1c levels above 8%, a negative correlation was observed between plasma 8-OHdG concentrations and total cholesterol levels. Importantly, none of these subgroup associations remained statistically significant after adjustment for multiple comparisons using the Benjamini–Hochberg procedure. Therefore, these findings should be interpreted as exploratory and do not provide conclusive evidence of associations within the individual HbA1c subgroups. Similarly, Abudawood et al. observed increased serum 8-OHdG concentrations in patients with T2DM compared with healthy individuals but did not find a significant association between oxidative DNA damage and HbA1c levels [32]. The absence of a clear association between plasma 8-OHdG concentrations and HbA1c levels in our study, despite higher 8-OHdG concentrations in patients with T2DM than in controls, may reflect differences in the biological processes captured by these measures. HbA1c reflects average glycaemic exposure over the preceding approximately 2–3 months, whereas plasma 8-OHdG concentrations may be influenced by oxidative DNA damage and DNA repair processes occurring over different time scales. This possibility remains hypothetical and requires further investigation. In contrast, other studies have reported positive associations between 8-OHdG levels and HbA1c [29,33,34].

Beyond its relationship with metabolic control, increasing evidence suggests that 8-OHdG may also be associated with the development and progression of chronic complications of diabetes. Thomas et al. reported that higher 8-OHdG concentrations were associated with the severity of cardiovascular and renal complications, as well as with an increased risk of mortality in patients with T2DM [35]. Similarly, Xu et al. observed increased urinary 8-OHdG levels in patients with diabetic nephropathy, particularly in those with microalbuminuria, supporting its potential utility as a marker of disease progression [36]. Moreover, higher urinary 8-OHdG levels have been positively correlated with proteinuria in patients with diabetic nephropathy and with high-risk (moderate-to-severe) diabetic retinopathy, two major microvascular complications of diabetes mellitus [37,38]. Experimental evidence also provides support for this hypothesis. Ishibashi et al. demonstrated the accumulation of 8-OHdG in retinal tissue in a mouse model of diabetic retinopathy, supporting the involvement of oxidative stress in the development of diabetic retinopathy [39]. Consistent with these findings, Dong et al. suggested that urinary 8-OHdG measurement may represent a useful tool for the early detection and monitoring of diabetic retinopathy, although further studies are needed to establish its diagnostic and prognostic value [29]. In our study, plasma 8-OHdG concentrations were not significantly correlated with eGFR among patients with T2DM.

The available evidence suggests an association between oxidative DNA damage and the pathophysiology of T2DM and its chronic complications. Among the biomarkers currently investigated, 8-OHdG has emerged as one of the most studied indicators of oxidative DNA damage. Its measurement in biological fluids may complement conventional clinical and biochemical assessments and may provide an early indication of oxidative stress and metabolic alterations associated with T2DM [19,40].

The present study has several limitations that should be considered when interpreting the findings. First, the cross-sectional design of the study does not allow causal relationships between plasma 8-OHdG concentrations and the investigated parameters to be established. Second, the relatively small sample size, particularly after stratification into HbA1c subgroups, may have reduced the statistical power to detect modest associations. Although renal function was assessed using eGFR in patients with T2DM, eGFR was not available for the healthy controls and therefore could not be evaluated as a potential confounding factor in the between-group comparison. In addition, several factors known or potentially relevant to 8-OHdG concentrations, including diabetes duration, hypertension, diabetic complications, inflammatory status, lifestyle-related factors, smoking status, body-mass index (BMI), and other markers of oxidative injury, were not systematically evaluated. Comprehensive information on pharmacological treatment was also not available for all participants, precluding a systematic evaluation of the potential effects of antidiabetic, lipid-lowering, antihypertensive, and other concomitant therapies on plasma 8-OHdG concentrations. Consequently, the potential contribution of these factors cannot be excluded. Furthermore, the control group was not fully matched to the T2DM group for age, and the observed age difference may have introduced residual confounding despite the adjustment for age using ANCOVA. In addition, the control group was selectively composed of metabolically healthy individuals, and the ROC cutoff was derived and evaluated in the same relatively small cohort, without independent validation. These factors may have led to an overestimation of the apparent discriminatory performance of 8-OHdG, and the ROC findings should therefore be considered exploratory. Finally, the study was conducted in a relatively limited clinical population. Correlations observed in the overall cohort were therefore interpreted cautiously, as associations that were not replicated within the T2DM group may partly reflect between-group differences rather than associations specific to T2DM. Further studies with larger sample sizes and more comprehensive clinical and treatment data are required to validate these observations.

Despite these limitations, the study provides additional evidence of higher plasma 8-OHdG concentrations in patients with T2DM than in controls, although the magnitude of this difference was attenuated after adjustment for age. No significant differences were observed across HbA1c categories. In the overall study population, plasma 8-OHdG concentrations were associated with age, fasting blood glucose, and triglyceride levels, suggesting a potential relationship between oxidative DNA damage and metabolic alterations. Further large-scale prospective studies are required to standardize analytical methodologies, establish clinically relevant reference values, and define the role of 8-OHdG in routine clinical practice.

## 5. Conclusions

The present study found higher plasma 8-OHdG concentrations in patients with T2DM than in healthy controls, supporting an association between oxidative DNA damage and T2DM. However, the between-group difference was smaller after adjustment for age, highlighting the need for caution when interpreting this association. Plasma 8-OHdG concentrations were associated with age, fasting blood glucose, and triglyceride levels in the overall population. No significant differences in plasma 8-OHdG concentrations were observed across HbA1c subgroups. Overall, these findings suggest that plasma 8-OHdG may reflect oxidative DNA damage associated with T2DM. However, larger prospective studies are required to validate these observations and further clarify the clinical significance of plasma 8-OHdG measurement, particularly its potential utility for risk assessment and disease monitoring.

## Figures and Tables

**Table 1:** Biochemical characteristics of the study population divided according to the presence/absence of T2DM.

	All Patients (*n* = 91)	Patients with T2DM (*n* = 50)	Controls (*n* = 41)
Age (years)	60.6 ± 10.7	68 ± 8.36	51.6 ± 4.6
Fasting blood glucose (mg/dL)	126.09 ± 48	153.52 ± 49.76	92.64 ± 7.95
Triglycerides (mg/dL)	125.71 ± 53.49	147.21 ± 53.67	99.49 ± 40.34
Cholesterol (mg/dL)	180.21 ± 43.5	164.75 ± 48.76	199.07 ± 26.13
HDL cholesterol (mg/dL)	54.01 ± 15.11	48.46 ± 11.52	60.78 ± 16.3
LDL cholesterol (mg/dL)	109.53 ± 38.41	96.06 ± 42.79	125.96 ± 23.95
8-OHdG (ng/mL)	70.55 ± 50.4	92.41 ± 58.04	43.90 ± 16.26

**Table 2:** Comparative analysis of 8-OHdG levels in patients with T2DM and controls.

8-OHdG (ng/mL)							IQR		Welch’s *t*-Test
	N	Mean	SD	Min	Max	Median	Q1	Q3	
T2DM	50	92.41	58.04	12.20	216.74	88.63	38.28	142.80	t = 5.645
Controls	41	43.90	16.26	16.58	76.47	41.52	32.01	55.61	*p* < 0.001
	Cohen’s d = 1.092/95% CI = (0.646–1.532)								
Total	91	70.55	50.40	12.20	216.74	47.04	32.41	96.53	

N = number of samples; SD = standard deviation; Min = minimum; Max = maximum; Q1 = first quartile; Q3 = third quartile; IQR = interquartile range.

**Table 3:** Correlations between 8-OHdG concentrations and age, fasting blood glucose, and lipid profile parameters in the overall study population and patients with T2DM.

	All Patients				T2DM Patients			
8OHdG vs.	ρ	*p* Value	95% CI		ρ	*p* Value	95% CI	
			LL	UL			LL	UL
Age (years)	0.379	<0.001	0.164	0.556	0.233	0.104	−0.081	0.526
Fasting blood glucose (mg/dL)	0.266	0.011	0.059	0.474	−0.069	0.634	−0.355	0.220
Triglycerides (mg/dL)	0.376	<0.001	0.178	0.554	0.300	0.034	−0.027	0.613
Cholesterol (mg/dL)	−0.175	0.097	−0.389	0.052	−0.121	0.403	−0.405	0.198
HDL cholesterol (mg/dL)	−0.171	0.106	−0.369	0.035	−0.119	0.409	−0.394	0.196
LDL cholesterol (mg/dL)	−0.181	0.086	−0.406	0.052	−0.111	0.443	−0.376	0.202

LL—lower limit; UL—upper limit. 95% confidence intervals (CIs) for Spearman’s correlation coefficients were estimated using bootstrap resampling.

**Table 4:** Correlations between 8-OHdG concentrations and laboratory parameters across HbA1c subgroups of patients with T2DM.

	HbA1c < 7%			HbA1c Levels of 7–8%			HbA1c > 8%		
8OHdG vs.	ρ	*p*-Value	q	ρ	*p*-Value	q	ρ	*p*-Value	q
Age (years)	0.089	0.692	0.952	0.625	0.030	0.228	0.220	0.412	0.760
Blood sugar (mg/dL)	0.025	0.913	0.952	−0.308	0.330	0.66	−0.279	0.295	0.643
HbA1c (%)	0.040	0.859	0.952	−0.401	0.196	0.470	−0.347	0.187	0.470
Triglycerides (mg/dL)	0.551	0.008	0.192	0.490	0.106	0.37	−0.047	0.863	0.952
Cholesterol (mg/dL)	0.446	0.038	0.228	−0.154	0.633	0.952	−0.538	0.031	0.228
HDL cholesterol (mg/dL)	0.013	0.954	0.954	−0.575	0.050	0.24	0.040	0.884	0.952
LDL cholesterol (mg/dL)	0.324	0.142	0.426	−0.056	0.863	0.952	−0.415	0.110	0.377
eGFR (mL/min/1.73 m^2^)	0.025	0.913	0.952	−0.091	0.779	0.952	−0.194	0.471	0.807

ρ, Spearman’s correlation coefficient; *p*, unadjusted *p*-value; q, Benjamini–Hochberg-adjusted *p*-value controlling the false discovery rate (FDR).

## Data Availability

The original contributions presented in this study are included in the article. Further inquiries can be directed to the corresponding author.

## Abbreviations

The following abbreviations are used in this manuscript:

T2DM	Type 2 diabetes mellitus
ROS	Reactive oxygen species
8OHdG	8-Hydroxy-2′-deoxyguanosine
ELISA	Enzyme-linked immunosorbent assay
eGFR	Estimated glomerular filtration rate
HbA1c	Glycated haemoglobin
8-oxo-dG	8-Oxo-7,8-dihydro-2′-deoxyguanosine
CIs	confidence intervals
ROC	Receiver operating characteristic
A	Adenine
T	Thymine
C	Cytosine
EDTA	Ethylene diamine tetraacetic acid
FDR	False discovery rate
G	Fasting blood glucose
TG	Triglycerides
TC	Total cholesterol
HDL-C	HDL-cholesterol
LDL-C	LDL-cholesterol
HPLC	High-performance liquid chromatography
OD	Optical density
SD	Standard deviation
AUC	Area under the curve
